# Detection of arterial remodeling using epicardial adipose tissue assessment from CT calcium scoring scan

**DOI:** 10.3389/fcvm.2025.1543816

**Published:** 2025-03-14

**Authors:** Juhwan Lee, Tao Hu, Michelle C. Williams, Ammar Hoori, Hao Wu, Justin N. Kim, David E. Newby, Robert Gilkeson, Sanjay Rajagopalan, David L. Wilson

**Affiliations:** ^1^ Department of Biomedical Engineering, Case Western Reserve University, Cleveland, OH, United States; ^2^ BHF Centre for Cardiovascular Science, University of Edinburgh, Edinburgh, United Kingdom; ^3^ Harrington Heart and Vascular Institute, University Hospitals Cleveland Medical Center, Cleveland, OH, United States; ^4^ Department of Radiology, Case Western Reserve University, Cleveland, OH, United States

**Keywords:** high risk plaque features, positive remodeling, computed tomography calcium scoring, fat-omics, machine learning, classification, major adverse cardiovascular events

## Abstract

**Introduction:**

Non-contrast CT calcium scoring (CTCS) exams have been widely used to assess coronary artery disease. However, their clinical applications in predicting coronary arterial remodeling remain unknown. This study aimed to develop a novel machine learning model to predict positive remodeling (PR) from CTCS scans and evaluate its clinical value in predicting major adverse cardiovascular events (MACE).

**Methods:**

We analyzed data from 1,324 patients who underwent both CTCS and CT angiography. PR was defined as an outer vessel diameter at least 10% greater than the average diameter of the segments immediately proximal and distal to the plaque. We utilized a total of 246 features, including 23 clinical features, 12 Agatston score-derived features, and 211 epicardial fat-omics features to predict PR. Feature selection was performed using Elastic Net logistic regression, and the selected features were used to train a CatBoost machine learning model. Classification performance was evaluated using 1,000 repetitions of five-fold cross-validation and survival analyses, comparing actual and predicted PR in the context of predicting MACE.

**Results:**

PR was identified in 429 patients (32.4%). Using Elastic Net, we identified the top 13 features, including four clinical features, three Agatston score-derived features, and six fat-omics features. Our method demonstrated excellent classification performance for predicting PR, achieving a sensitivity of 80.3 ± 1.7%, a specificity of 89.7 ± 1.7%, and accuracy of 81.9 ± 2.5%. The Agatston-score-derived and fat-omics features provided additional benefits, improving classification performance. Furthermore, our model effectively predicted MACE, with a hazard ratio (HR) of 4.5 [95% confidence interval (CI): 3.2–6.4; C-index: 0.578; *p* < 0.00001] in the training set and an HR of 3.2 (95% CI: 2.5–4.0; C-index: 0.647; *p* < 0.00001) in the external validation set.

**Conclusion:**

We developed an innovative machine learning model to predict coronary arterial remodeling from epicardial fat and calcification features from low-cost/no-cost screening CTCS scans. Our results suggest that vast number of CTCS scans can support more informed clinical decision-making and potentially reduce the need for invasive and costly testing for low-risk patients.

## 1 Introduction

Coronary artery disease (CAD) is the leading cause of morbidity and mortality in industrialized countries globally (1, 2), highlighting the urgent need for advanced diagnostic tools to predict and mitigate its progression at an early stage. Early detection and accurate prediction of CAD are crucial for timely intervention and improving patient outcomes. Various imaging techniques have been employed to visualize plaque in coronary vessels and characterize CAD. Among these techniques, coronary computed tomography angiography (CCTA) is considered the most effective for identifying the presence and distribution of CAD (3).

CCTA can identify high-risk plaque features such as low-attenuation plaque, napkin-ring sign, spotty calcification, and positive remodeling (PR), which are indicators of increased risk for cardiovascular events. PR refers to the outward expansion of a coronary artery in response to atherosclerotic plaque buildup, and it has been shown to be a strong indicator of major adverse cardiovascular events (MACE) in several studies (4–8). However, the use of CCTA is often limited by factors such as cost, variability in assessments, the need for contrast agents, and exposure to ionizing radiation. Additionally, the relationship between PR and the likelihood of adverse events is not fully understood.

Non-contrast CT calcium scoring (CTCS) exam can provide direct evidence of coronary atherosclerosis through the detection of calcifications in the coronary arteries. It is recognized by several guidelines as a preferred tool for risk assessment (9, 10). However, to date, no studies have investigated whether the use of non-contrast CTCS could predict high-risk plaque features. While CTCS is well-established for assessing cardiovascular risk, its potential to predict specific disease conditions is not fully utilized, presenting an opportunity to expand its diagnostic capabilities. We hypothesize that quantitative measurements from CTCS are related to high-risk plaque features. Specifically, we identified features in CTCS images associated with the presence of PR. This analysis aims to uncover significant features from CTCS relevant to arterial remodeling and enable further correlative studies with CCTA to identify obstructive disease based on CTCS findings. In this regard, we have developed novel feature sets using CTCS, such as calcium-omics (11) and fat-omics (12), which have shown strong associations with future MACE outcomes. Given that CTCS offers unique perspective on coronary plaque, there is a strong rationale to relate CTCS findings to cardiovascular risk assessed by CCTA.

In this study, we developed a novel machine learning model to predict the presence of PR from low-cost or no-cost screening non-contrast CTCS scans and evaluated its performance using extensive multi-center datasets. We analyzed various clinical features, Agatston score-derived features, and epicardial fat-omics features (12) to identify the most relevant ones. We then trained multiple machine learning models and compared their performance in predicting PR and MACE.

## 2 Methods

### 2.1 Study design and patient population

This research was conducted as a sub-study within the Scottish COmputed Tomography of the HEART (SCOT-HEART) multicenter randomized controlled trial (ClinicalTrials.gov. Unique identifier: NCT01149590). The primary trial received approval from the local ethics committee, and all participants provided written informed consent. This specific sub-study was carried out under a data use agreement between the University of Edinburgh, Edinburgh, UK, and the University Hospitals Cleveland Medical Center, Cleveland, Ohio, USA. The main findings of the primary study have been published previously (13–15). In summary, of the 4,146 participants who attended the cardiology outpatient clinic, 2,073 were assigned to the intervention group. Among them, 1,778 underwent CT scans, and 1,324 of these scans were of sufficient quality for analysis in this sub-study. Further details can be found in other publications (7, 16).

### 2.2 CT image acquisition

All participants underwent both non-contrast electrocardiogram-gated CTCS and contrast-enhanced electrocardiogram-gated CCTA. The imaging was conducted using either 64- or 320-detector row scanners (Brilliance 64 from Philips Medical Systems, Netherlands; Biograph mCT from Siemens, Germany; Aquilion ONE from Toshiba Medical Systems, Japan). Adjustments for tube current, voltage, and the volume of iodine-based contrast agent were made according to each participant's body mass index (BMI).

### 2.3 Assessment of positive remodeling

**Figure 1:**
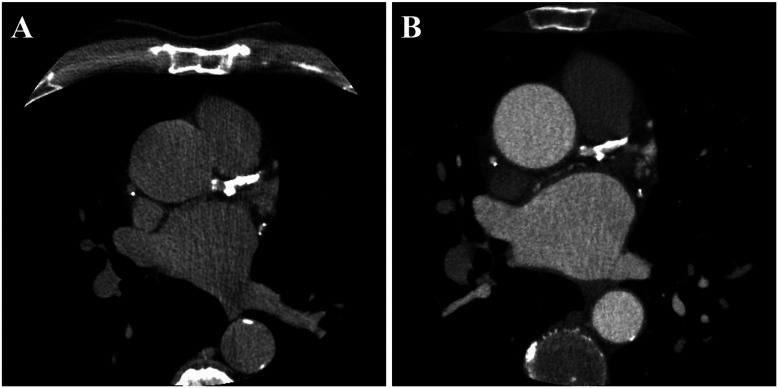
A representative example of paired CTCS **(A)** and CCTA **(B)** images showing the presence of PR. PR was observed in all major coronary arteries, including the left anterior descending (LAD), left circumflex (LCX), and right coronary artery (RCA). The total Agatston score was 3,027, distributed as follows: left main, 43; LAD, 1,168; LCX, 633; and RCA, 1,183.

The assessment of positive remodeling for each coronary segment was conducted using standardized semi-automated software (Autoplaque, Version 2.5, Cedars-Sinai Medical Center). PR was characterized by an outer vessel diameter that was at least 10% greater than the average diameter of the segments directly proximal and distal to the plaque (remodeling index >1.1) (17). The remodeling index was calculated by semi-automatically extracting coronary artery centerlines and identifying the vessel lumen, wall, and plaque components on multi-planar reformatted images, with manual adjustments as necessary. Subsequently, the vessel diameters were measured to determine the remodeling index. In this study, PR was evaluated in the proximal, mid, and distal regions of each major coronary artery, including the left anterior descending (LAD), left circumflex (LCX), and right coronary artery (RCA). A patient was considered to have PR if at least one PR was present in any of these arteries. The reliability of observer agreement in assessing positive remodeling has generally been reported as fair (18). Figure 1 illustrates an example of paired CTCS and CCTA images showing PR in the left anterior descending artery.

### 2.4 Feature extraction

We examined 23 clinical, 12 Agatston score-derived, and 211 fat-omics features to predict the presence of PR.

(1) *Clinical features:* These included baseline characteristics (e.g., gender, BMI, age, cholesterol, family history, etc.), blood tests (e.g., cholesterol levels), medications (e.g., statin, ACE inhibitor, and Beta blocker), and more. Table 1 lists the clinical features analyzed as predictors of PR.
(2) *Agatston score-derived features:* These included the Agatston scores for all coronary arteries, the logarithms of the Agatston scores (with 1 added to avoid infinite values), the diffusivity index (19), and high coronary artery calcification (CAC). The diffusivity index was calculated as 1 min the ratio of the Agatston score of the most affected vessel to the total Agatston score, indicating the distribution of coronary artery calcification. A higher diffusivity index signifies a more diffuse distribution, while a lower index indicates that the calcification is concentrated in a single artery. High CAC was defined as positive when the total Agatston score exceeded 1,000. Table 2 lists the Agatston score-derived features.
(3) *Fat-omics features:* We analyzed 211 handcrafted, pathophysiological-inspired fat-omics features, divided into morphological, intensity, and spatial categories (12). Using our previously developed DeepFat method (20), we segmented the epicardial adipose tissue regions within the pericardium. Morphological features included measurements such as volume, principal axis lengths, and epicardial fat thickness. Intensity features included statistical metrics like the minimum, maximum, and mean Hounsfield units (HU), skewness, and histogram bins. For spatial analysis, we divided the heart area into four equally thick slabs of image slices from top to bottom and four equidistant ribbons from the outer to inner regions. Detailed descriptions of fat-omics features are provided elsewhere (12).

**Table 1:** Baseline clinical characteristics of PR (+) and PR (−) groups.

Features	PR (+) (*n* = 429)	PR (−) (*n* = 895)	*p*-value
Male	316/429 (73.7%)	421/895 (47.0%)	<0.00001
BMI	28.6 ± 4.7	30.1 ± 5.9	<0.00001
Age	60.5 ± 8.0	55.7 ± 9.6	<0.00001
BMI band (≥30 or <30)	142/429 (33.1%)	392/895 (43.8%)	0.0002
Age band (18–59 or 60–75)	239/429 (55.7%)	338/895 (37.8%)	<0.00001
Diabetes Mellitus	53/429 (12.4%)	95/895 (10.6%)	0.35
Height	1.72 ± 0.09	1.69 ± 0.10	<0.00001
Weight	84.1 ± 15.1	86.0 ± 18.9	0.08
Cigarettes per day	2.9 ± 6.6	2.8 ± 7.3	0.77
Hypertension	171/424 (40.3%)	288/886 (32.5%)	0.005
Total cholesterol	5.04 ± 1.94	5.02 ± 1.82	0.86
HDL cholesterol	0.92 ± 0.66	1.06 ± 0.70	0.0006
CHD family history	190/425 (44.7%)	392/889 (44.3%)	0.90
Systolic blood pressure	140.4 ± 21.5	137.3 ± 25.0	0.03
Diastolic Blood Pressure	81.4 ± 12.0	81.1 ± 14.5	0.74
Chest pain (anginal vs. non-anginal)	315/429 (73.4%)	479/895 (53.5%)	<0.00001
Antiplatelet	360/429 (83.9%)	441/895 (49.3%)	<0.00001
Statin	343/429 (80.0%)	394/895 (44.0%)	<0.00001
Ace inhibitor	104/429 (24.2%)	126/895 (14.1%)	<0.00001
Calcium blocker	50/429 (11.7%)	75/895 (8.4%)	0.057
Nitrates	158/429 (36.8%)	177/895 (19.8%)	<0.00001
Betablocker	215/429 (50.1%)	248/895 (27.7%)	<0.00001
Hyperlipidemia	321/429 (74.8%)	481/895 (53.7%)	0.001

PR, positive remodeling.

**Table 2:** Agatston score-derived features of PR (+) and PR (−) groups.

Features	PR (+) (*n* = 429)	PR (−) (*n* = 895)	*p*-value
LM Agatston Score	30.8 ± 84.1	4.1 ± 19.9	<0.00001
LAD Agatston Score	260.9 ± 324.8	49.4 ± 211.4	
LCX Agatston Score	117.7 ± 289.0	21.5 ± 165.6	
RCA Agatston Score	243.7 ± 559.9	31.4 ± 178.9	
Total Agatston Score	653.2 ± 967.5	106.4 ± 480.2	
Log (LM Agatston Score)	0.57 ± 0.84	0.13 ± 0.43	
Log (LAD Agatston Score)	2.02 ± 0.75	0.55 ± 0.87	
Log (LCX Agatston Score)	1.17 ± 1.03	0.26 ± 0.63	
Log (RCA Agatston Score)	1.46 ± 1.10	0.35 ± 0.71	
Log (total Agatston score)	2.38 ± 0.74	0.75 ± 0.98	
High CAC	90/429 (21.0%)	19/895 (2.1%)	
Diffusivity index	0.30 ± 0.21	0.08 ± 0.16	

LM, left main; LAD, left anterior descending; LCX, left circumflex; RCA, right coronary artery; PR, positive remodeling.

### 2.5 Feature selection

We employed Elastic Net regression (21), which integrates the properties of both Lasso (L1) and Ridge (L2) regularization, for feature selection in our classification models. The mixing parameter (α) was set to 0.5 to equally balance the effects of L1 and L2 regularization. A series of 100 regularization values (λ) was automatically generated on a logarithmic scale, ranging from a maximum value that results in all coefficients being zero to a minimum value. To optimize these hyperparameters and avoid overfitting, we utilized 5-fold cross-validation. During this process, the data was divided into five subsets, with the model iteratively trained on four subsets and validated on the remaining one. This approach ensured the robustness and generalizability of the model to new data.

### 2.6 Machine learning model

After identifying the optimal features with Elastic Net regression, we trained a machine learning model using the CatBoost algorithm (22). We optimized the CatBoost model through hyperparameter tuning using a grid search. The hyperparameters tuned included the number of iterations (100), learning rate (0.01), depth (6), L2 leaf regularization (3), random subspace method (0.75), border count (64), loss function (logloss), evaluation metric (F1), bootstrap type (Bernoulli), subsample (0.6), and thread count (1). This grid search enabled us to find the best combination of hyperparameters for our dataset. The hyperparameter search ranges for each model are provided in Supplementary Table S1.

We developed and trained three different models with varying feature combinations: (*Model 1*) clinical features only, (*Model 2*) clinical features plus Agatston score-derived features, and (*Model 3*) clinical features, Agatston score-derived features, and fat-omics features. Each model underwent the same hyperparameter optimization process. All the employed codes, including DeepFat (20), Elastic Net regression for feature selection, and CatBoost machine learning models, are available in our GitHub repository (https://github.com/Thefreak123TH).

### 2.7 Performance evaluation

To ensure the robustness and reliability of our model, we employed 1,000 iterations of 5-fold cross-validation. This method involved dividing the dataset into five subsets, training the model on four subsets, and validating it on the remaining subset. Repeating this process 1,000 times helped to average out variability and prevent overfitting. The classification performance was evaluated using conventional metrics such as sensitivity, specificity, accuracy, and area under the receiver operating characteristic curve (AUC).

To investigate performance variance, we compared the optimized CatBoost model with three other established methods: Support Vector Machine (SVM) (23), Random Forest (RF) (24), and XGBoost (25). Detailed training information for all machine learning models is provided in Supplementary Table S1. Each model was trained and tested using the same dataset split to ensure a fair comparison. This comprehensive evaluation allowed us to identify the most effective machine learning approach for our classification task.

In addition to conventional metrics, we conducted clinically relevant assessments to evaluate the model's utility in predicting MACE. Following classification, we used Kaplan–Meier survival analysis to estimate survival probabilities and stratify patients based on predicted and actual PRs. Cox proportional hazards models were then fitted using the predicted and actual PRs as covariates to assess their association with MACE. The survival predictions derived from these analyses were compared for consistency and clinical relevance. MACE was defined as a composite outcome of nonfatal myocardial infarction, nonfatal stroke, cardiovascular death, and revascularization. For external validation, we used a subset of the CLARIFY trial (ClinicalTrials.gov. Unique identifier: NCT04075162), comprising 2,316 patients who underwent both CCTA and CTCS.

### 2.8 Statistical analysis

Continuous variables were presented as mean ± standard deviation, and categorical variables were reported as frequencies. Comparisons between PR and non-PR groups were made using Student's *t*-test for continuous variables and the Chi-square test for categorical variables. Normality of continuous variables was assessed using the Shapiro–Wilk test. To evaluate the models' performance, we used McNemar's test (for sensitivity and specificity) and the Delong test (for AUC) to compare *Model 1* with *Model 2* and *Model 2* with *Model 3*. To predict MACE, we utilized Kaplan–Meier survival analysis and Cox proportional hazard modeling, reporting hazard ratios (HR) and 95% confidence intervals (CI). A *p*-value of less than 0.05 was considered statistically significant. All statistical analyses were conducted using R Studio software (version 2024.04.1, R Foundation for Statistical Computing, Vienna, Austria).

## 3 Results

Among the 1,324 patients, PR was identified in 429 patients (32.4%). The PR group had a significantly higher prevalence of males, hypertension, chest pain, hyperlipidemia, and medications (except for calcium blockers) than the non-PR group (*p* < 0.05). Additionally, age, height, and systolic blood pressure were significantly higher in PR group compared to the non-PR group (*p* < 0.05). While there was no significant difference in total cholesterol levels, HDL cholesterol was significantly lower in the PR group (*p* < 0.05). The baseline characteristics of the study population, comparing the PR and non-PR groups, are detailed in Table 1. All the Agatston score-derived features were significantly higher in the PR group compared to the non-PR group (Table 2).

**Figure 2:**
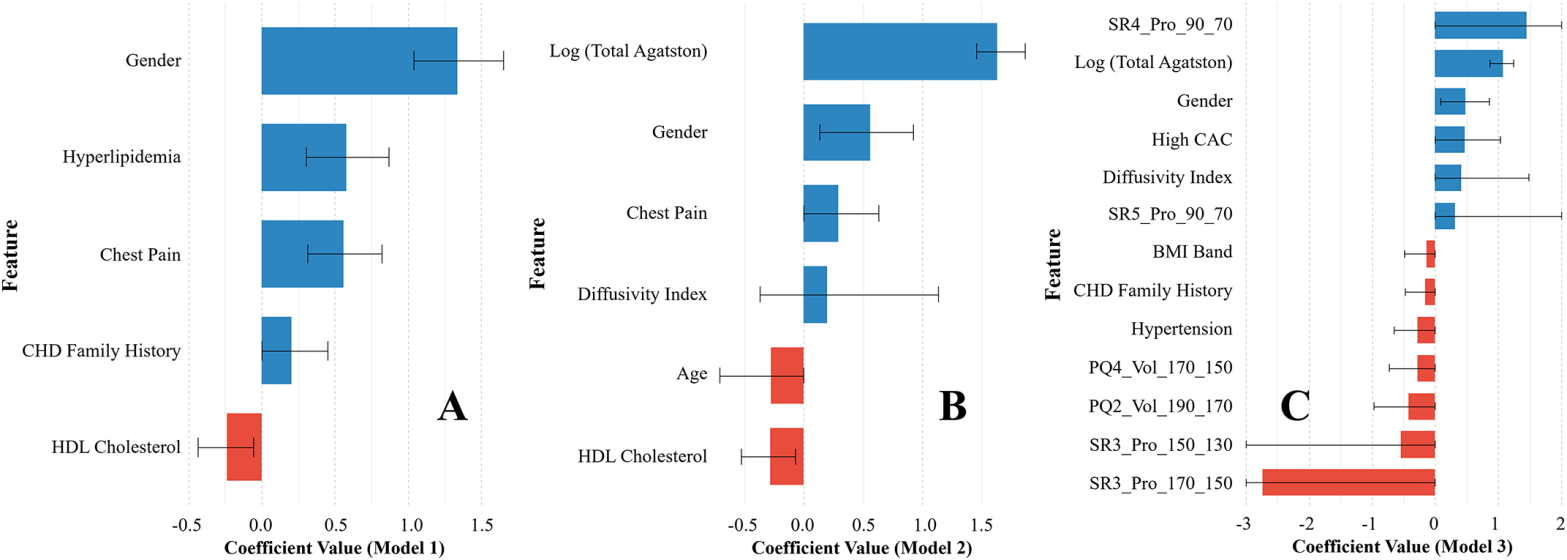
Variable importance plots of the three prediction models [**(A)** *Model 1*, **(B)** *Model 2*, and **(C)** *Model 3*] selected by Elastic Net regression. For Model 3, 13 features were selected and used to train the CatBoost machine learning classification model. The 95% confidence interval (CI) is provided for each feature. The actual CI ranges for SR4_Pro_90_70, SR5_Pro_90_70, SR3_Pro_150_130, and SR3_Pro_170_150 were clipped for better visualization.

We selected the top 13 features for *model 3* using Elastic Net regression, which included four clinical features, three Agatston score-derived features, and six fat-omics features (Figure 2C). Among them, the top three were fat-omics (SR4_Pro_90_70, SR3_Pro_170_150, and SR3_Pro_150_130), highlighting the significant impact of epicardial fat assessment. Notably, all fat-omics features were derived from histograms of HU values (Supplementary Figure S1 and Table S2). Such assessments are related to fat inflammation, thought to be a driver of cardiovascular risk (see Discussion). The best features for *Models 1* and *2* were also determined by Elastic Net (Figures 2A,B).

Our method (*Model 3*) demonstrated excellent classification performance for predicting PR, achieving a sensitivity of 80.3 ± 1.7%, a specificity of 89.7 ± 1.7%, an accuracy of 81.9 ± 2.5%, and an AUC of 88.7 ± 1.2% (Table 3). *Model 1* had the poorest classification performance, with the lowest values across all metrics. The classification performance improved significantly with the addition of Agatston score-derived features (*Model 2*) (*p* < 0.05). Adding fat-omics to *Model 2* further improved sensitivity by 10% (from 70.0% to 80.3%) and specificity by 4% (from 85.9% to 89.7%) in *Model 3* (*p* < 0.05). Among the four machine learning methods employed, the CatBoost method exhibited the best classification performance (Table 4). Although SVM showed higher sensitivity with much smaller standard deviation (87.9 ± 0.4%), its specificity was very low (67.9 ± 1.0%) compared to other methods. RF and XGBoost methods demonstrated similar classification results. The classification results of the *Models 1–2* obtained using the other machine learning methods are provided in Supplementary Tables S3, S4.

**Table 3:** Classification results of each model obtained using 1,000 repeated 5-fold cross validation.

Model	Sensitivity	Specificity	Accuracy	AUC
Model 1 (Clinical)	46.3 ± 5.1	76.3 ± 3.1	71.2 ± 2.7	70.5 ± 3.1
Model 2 (Clinical + Agatston score)	70.0 ± 4.6	85.9 ± 2.3	81.6 ± 2.0	88.4 ± 1.8
Model 3 (Clinical + Agatston score + Fat-omics)	80.3 ± 1.7	89.7 ± 1.7	81.9 ± 2.5	88.7 ± 1.2

The exact same training and testing data were used for each model, with hyperparameter optimization using grid search.

**Table 4:** Comparison of the proposed method (CatBoost) against other established machine learning approaches, including random forest (RF), support vector machine (SVM), and XGBoost.

Model	Sensitivity	Specificity	Accuracy	AUC
RF	73.8 ± 3.5	87.2 ± 1.4	81.5 ± 2.0	87.8 ± 1.3
SVM	87.9 ± 0.4	67.9 ± 1.0	77.9 ± 0.6	88.0 ± 0.2
XGBoost	76.0 ± 4.7	87.9 ± 2.9	81.3 ± 2.0	88.0 ± 1.6
CatBoost	80.3 ± 1.7	89.7 ± 1.7	81.9 ± 2.5	88.7 ± 1.2

Results were obtained using 1,000 repeated five-fold cross validation. The exact same training and testing data were used for each method, with hyperparameter optimization using grid search.

**Figure 3:**
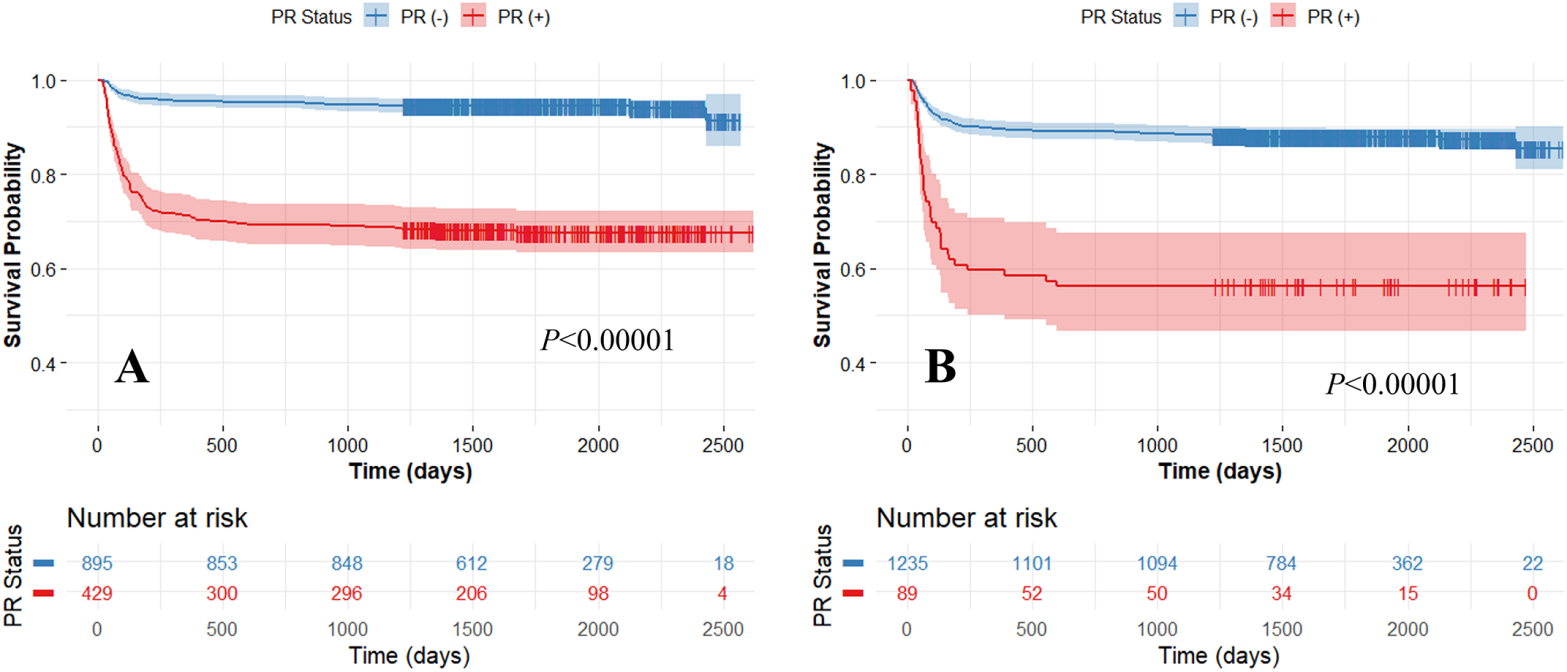
Kaplan-Meier survival curves between actual **(A)** and predicted (*Model 3*) **(B)** positive remodeling (PR) obtained using five-fold cross-validation. High and low-risk groups are split with the median risk. The x-axis represents survival time, and the y-axis represents the survival probability of patients. Both the actual and predicted PRs significantly differentiated between MACE and no-MACE groups (*p* < 0.00001). The colors are blue (no-MACE) and red (MACE).

**Figure 4:**
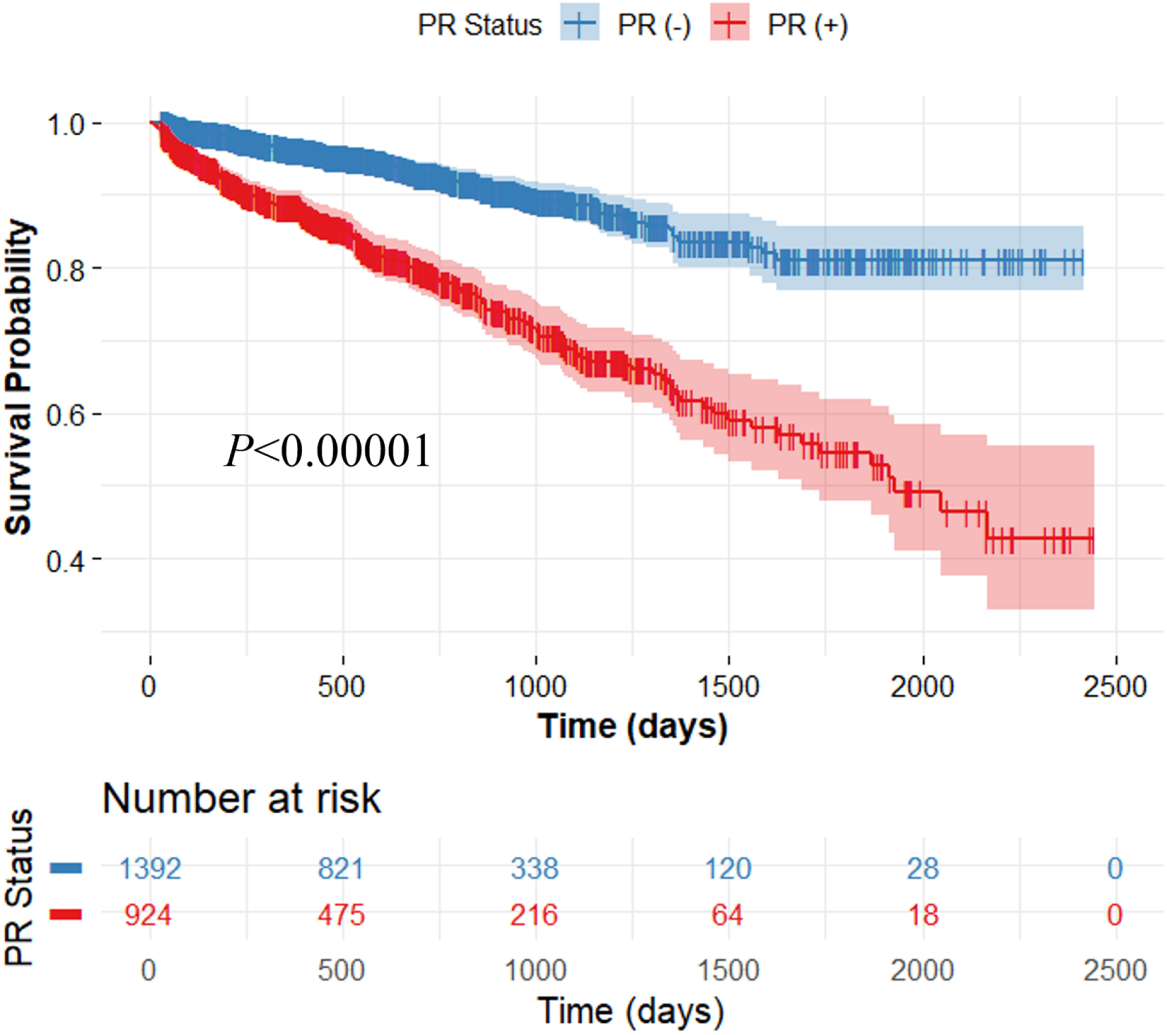
Kaplan-Meier survival curve on the external validation set consisting of 2,316 patients. For survival analysis, the CatBoost classification model (*Model 3*) was trained using the entire SCOT-HEART data (*n* = 1,324) and applied to the external validation set. The output of the trained model was used for survival analysis. The x-axis represents survival time, and the y-axis represents the survival probability of patients. The predicted positive remodeling (PR) significantly differentiated between MACE and no-MACE groups (*p* < 0.00001). The colors are blue (no-MACE) and red (MACE).

Our method demonstrated excellent prediction of MACE. In Kaplan–Meier analyses, both actual and predicted PR significantly differentiated between MACE and no-MACE groups (*p* < 0.00001) (Figure 3). However, the predicted PR showed a much wider range of 95% CI compared to the actual PR. In Cox proportional hazard analyses, the actual PR had a higher HR (6.5; 95% CI: 4.8–9.0; C-index: 0.722; *p* < 0.00001) than the predicted PR (4.5; 95% CI: 3.2–6.4; C-index: 0.578; *p* < 0.00001). When applied to the external validation set (CLARIFY, *n* = 2,316), our method significantly differentiated between MACE and no-MACE groups (*p* < 0.00001) (Figure 4), with an HR of 3.2 (95% CI: 2.5–4.0; C-index: 0.647; *p* < 0.00001).

## 4 Discussion

We built on our previous studies utilizing CT imaging (11, 12, 20, 26–31) and developed a novel machine learning model to predict the presence of PR from CTCS scans. To the best of our knowledge, this is the first study to examine CTCS imaging features as predictors of the arterial remodeling. The proposed study has several important contributions. (1) We utilized unique feature sets consisting of clinical, Agatston score-derived, and epicardial fat-omics features (12). The most relevant features were then determined using Elastic Net regression. (2) We implemented various state-of-the-art machine learning methods and compared their classification performance with different feature groups. This comprehensive evaluation allowed us to determine the most effective machine learning approach. (3) We further investigated the clinical value of the proposed method for predicting MACE using multi-center datasets (i.e., SCOT-HEART and CLARIFY).

Our fat-omics features had a significant impact on predicting PR, highlighting the importance of epicardial fat assessment. Epicardial fat is a visceral fat deposit located between the heart and the pericardium that can potentially cause local inflammation in the coronary arteries. This inflammation can directly contribute to coronary atherosclerosis and influence arterial remodeling, the process of structural changes in the coronary artery wall. Assessing epicardial fat is crucial because it serves as an active endocrine organ, secreting various adipokines and cytokines that can exacerbate inflammation and promote atherosclerotic changes. Additionally, the presence of epicardial fat has been associated with increased risk of plaque formation, arterial stiffening, and other adverse changes in the coronary arteries (32–34). In our previous study (12), we developed an automated method for the first time to quantitatively assess the epicardial fat deposition (fat-omics) and demonstrated the importance of elevated HU values for predicting MACE. Similarly, in this study, we found that the probabilities of EAT voxels with elevated HU values in the outer layers (e.g., SR4 and SR5) were potentially beneficial for prediction PR. The SR4 and SR5 regions correspond to areas where coronary arteries most likely located, which is why they were identified as strong predictors for PR (SR4_Pro_90_70 and SR5_Pro_90_70). The higher HU range (−90 to −70) is more indicative of lipid-rich necrotic cores, fibrotic-lipid mixed plaques, and inflamed pericoronary adipose tissue, all of which are strongly associated with PR. In contrast, the lower HU range (e.g., −170 to −150) is more reflective of adipose tissue, which may not directly contribute to PR. Inflammation and proteolysis promote outward expansion of the vessel wall, leading to PR. Since these processes are more strongly linked to regions with higher HU values, this explains their predictive importance.

The addition of Agatston score-derived features, particularly the logarithmic total Agatston score and diffusivity index, significantly impacted predicting PR. Higher Agatston scores indicate a greater burden of calcified plaque, reflecting more advanced and extensive coronary artery disease. This is associated with both positive and negative arterial remodeling, where the arterial wall undergoes structural changes in response to plaque accumulation. Positive remodeling can initially preserve the arterial lumen, while negative remodeling can lead to luminal narrowing and increased risk of cardiovascular events. Additionally, a higher diffusivity index indicates more diffuse distribution of calcification across the entire coronary arteries, suggesting that the spread of coronary calcification plays a crucial role in arterial remodeling. The diffuse calcification distribution may exacerbate the remodeling process, making it more challenging to manage and treat coronary artery disease effectively.

The CatBoost model demonstrated superior classification performance compared to other machine learning methods, including RF, SVM, and XGBoost. This enhanced performance can be attributed to several unique features of CatBoost. First, CatBoost is specifically designed to handle categorical features effectively without requiring extensive preprocessing or one-hot encoding, often necessary with other models. This results in better utilization of clinical features, Agatston score-derived features, and fat-omics features. Second, CatBoost employes an ordered boosting technique, which mitigates the prediction shift commonly encountered in traditional gradient boosting algorithms. This leads to more accurate and stable predictions, particularly compared to XGBoost. Additionally, CatBoost efficiently handles missing data and incorporates robust feature importance mechanisms, further enhancing its predictive capabilities. Its ability to manage data with varying distributions and complexities makes it particularly well-suited for medical imaging datasets, where such variability is common. In contrast, SVM showed the highest sensitivity in our experiments but suffered from very poor specificity, likely due to its tendency to overfit on positive instances, resulting in a higher false positive rate. Last, the model's inherent resistance to overfitting, combined with minimal hyperparameter tuning, helped improve classification performance.

Our method demonstrated high reproducibility, validated using extensive multi-center datasets, including those from the SCOT-HEART and CLARIFY trials. This evaluation across multiple centers is crucial as it confirms that the model performs robustly and is generalizable across various patient populations and imaging conditions. Although minor differences were observed in HR and C-index between the training and external validation sets (HR: 4.5, C-index: 0.578 vs. HR: 3.2, C-index: 0.647), our approach effectively predicted MACE (see Figures 3, 4). By employing datasets from different centers for training and testing, we minimized the risk of overfitting to a single-center dataset, enhancing the model's applicability in diverse settings. Our consistent classification performance across multiple independent cohorts suggests its reliability and potential for widespread clinical use in predicting arterial remodeling from CTCS data. Such rigorous validation is essential for establishing confidence in the model's predictions and ensuring its practical effectiveness in real-world clinical scenarios.

This study has a few limitations. First, the relatively small sample size may limit the generalizability of the findings, and results could vary with a larger cohort. Second, despite the full automation of the fat-omics calculations, each CTCS scan assessment required 15–20 min. Future improvements in software automation could streamline this process and enable wider use. Third, integrating additional quantified variables, such as calcium-omics (11), could potentially enhance predictive accuracy.

## 5 Conclusion

We developed an innovative machine learning model to predict coronary arterial remodeling from epicardial fat and calcification features from low-cost or no-cost screening non-contrast CTCS scans. The same model also predicted MACE. This suggests that the vast number of CTCS scans available can support more informed clinical decision-making and potentially reduce the need for invasive and expensive tests in low-risk patients.

## Data Availability

The original contributions presented in this study are included in the article/supplementary material. Further inquiries can be directed to the corresponding author.
