# Supplementary material for: Detection of arterial remodeling using epicardial adipose tissue assessment from CT calcium scoring scan
Source: Front Cardiovasc Med. 2025 Mar 14;12:1543816. doi: 10.3389/fcvm.2025.1543816 (PMC11951307; doi:10.3389/fcvm.2025.1543816)
Supplement: Supplementary file 1 [file Datasheet1.pdf]

Supplementary Material

1 Supplementary Figures and Tables

1.1 Supplementary Table

Supplementary Table S1. Training details of machine learning classification methods, including random forest (RF), support vector machine (SVM), XGBoost, and CatBoost. The hyperparameter search ranges for each model are also provided.

**RF**		**Searched Ranges**
Number of trees	500	100, 300, 500
m_{try}	4	2, 4, 6
Split rule	Gini	Gini
Minimum Node Size	5	3, 5, 7
**SVM**		
C	0.5	0.1, 0.1, 0.5
Sigma	0.064	0.001, 0.064, 0.01
Method	Radial	Linear, radial
Metric	ROC	Accuracy, ROC
Preprocess	Center	Center
**XGBoost**		
Number of rounds	50	50, 100, 150
Booster	gbtree	gbtree
ETA	0.1	0.01, 0.1
Gamma	0	0, 0.1
Maximum Depth	3	3, 6, 9
Minimum Child Weight	1	1, 3, 5
Subsample	0.6	0.6, 0.8
ColSample_bytree	0.75	0.5, 0.75
**CatBoost**		
Iterations	100	50, 100, 150
Learning Rate	0.01	0.001, 0.01, 0.1
Depth	6	3, 6, 9
L2_leaf_reg	5	3, 5
Random Subspace Method (RSM)	0.75	0.25, 0.5, 0.75
Border Count	64	32, 64
Loss Function	Log Loss	Log Loss
Evaluation Metric	F1	Log Loss, AUC, F1
Bootstrap Type	Bernoulli	Bernoulli
Subsample	0.6	0.6, 0.8
Thread Count	1	1, 5, 10

Supplementary Table S2. Six fat-omics features selected by Elastic Net regression for *Model 3*.

Features	Descriptions
SR4_Pro_90_70	In Spherical Region 4, Probability of EAT Voxels HU in range [-90,-70]
SR5_Pro_90_70	In Spherical Region 5, Probability of EAT Voxels HU in range [-90,-70]
PQ4_Vol_170_150	In PQ4, Volume of EAT Voxels HU in range [-170,-150]
PQ2_Vol_190_170	In PQ2, Volume of EAT Voxels HU in range [-190,-170]
SR3_Pro_150_130	In Spherical Region 3, Probability of EAT Voxels HU in range [-150,-130]
SR3_Pro_170_150	In Spherical Region 3, Probability of EAT Voxels HU in range [-170,-150]

Supplementary Table S3. Comparison of all machine learning approaches, including random forest (RF), support vector machine (SVM), XGBoost, and CatBoost, trained using clinical features only (model 1). Results were obtained using 1,000 repeated five-fold cross validation. The exact same training and testing data were used for each method, with hyperparameter optimization using grid search.

Methods	Sensitivity	Specificity	Accuracy	AUC
RF	36.2±5.6	74.3±1.4	71.3±1.6	69.6±2.6
SVM	87.0±0.7	36.1±2.3	61.5±0.9	69.4±0.5
XGBoost	38.5±5.9	74.6±3.4	71.1±2.8	70.4±3.2
CatBoost	46.3±5.1	76.3±3.1	71.2±2.7	70.5±3.1

Supplementary Table S4. Comparison of all machine learning approaches, including random forest (RF), support vector machine (SVM), XGBoost, and CatBoost, trained using clinical + Agatston score-derived features only (model 2). Results were obtained using 1,000 repeated five-fold cross validation. The exact same training and testing data were used for each method, with hyperparameter optimization using grid search.

Methods	Sensitivity	Specificity	Accuracy	AUC
RF	66.1±5.4	84.3±2.1	80.2±2.4	86.8±2.3
SVM	87.0±0.6	64.5±1.0	75.8±0.5	86.9±0.3
XGBoost	68.5±6.3	85.2±3.2	80.9±1.8	87.2±2.4
CatBoost	70.0±4.6	85.9±2.3	81.6±2.0	88.4±1.8

1.2 Supplementary Figures

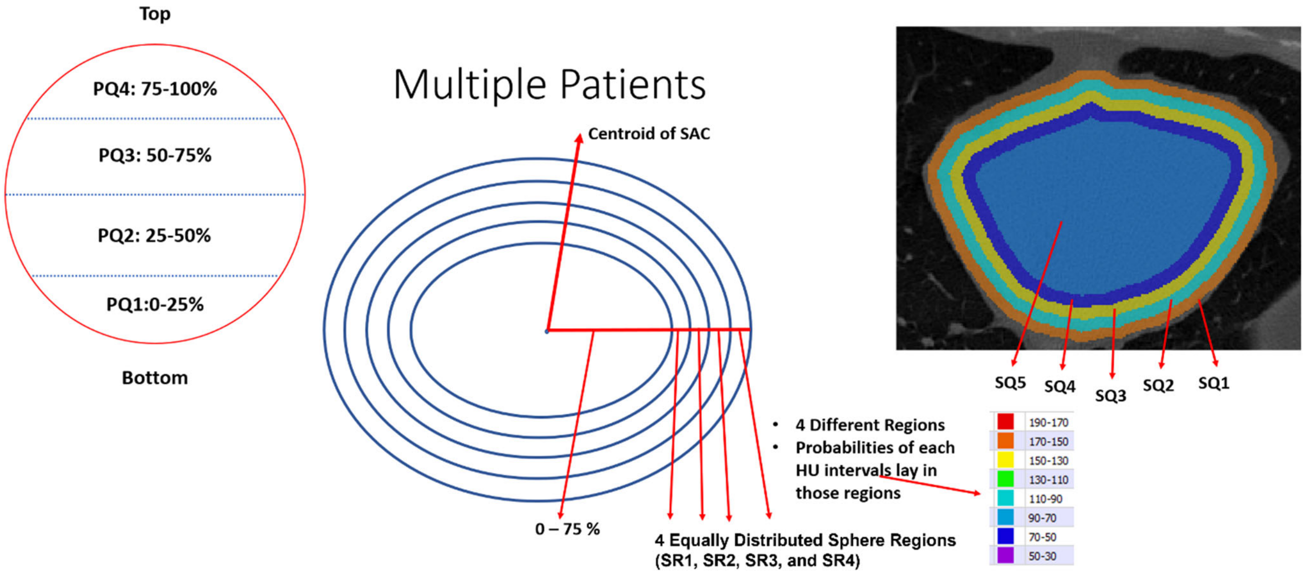

Supplementary Figure 1. Visualization of fat-omics features obtained from CTCS images.
